# Supplementary material for: Tead1a Initiates Transcriptional Priming Through the TEAD1a/YAP‐Notch1‐Spi1/Cebpα Axis to Promote Neutrophil Fate
Source: Adv Sci (Weinh). 2025 Aug 28;12(41):e05441. doi: 10.1002/advs.202505441 (PMC12591127; doi:10.1002/advs.202505441)
Supplement: Supplementary file 4 — Supplementary Dataset [file ADVS-12-e05441-s004.zip › 3-3.zip!/3-3/02-3.cluster/cluster8/Enrich/ego_BP.pdf]

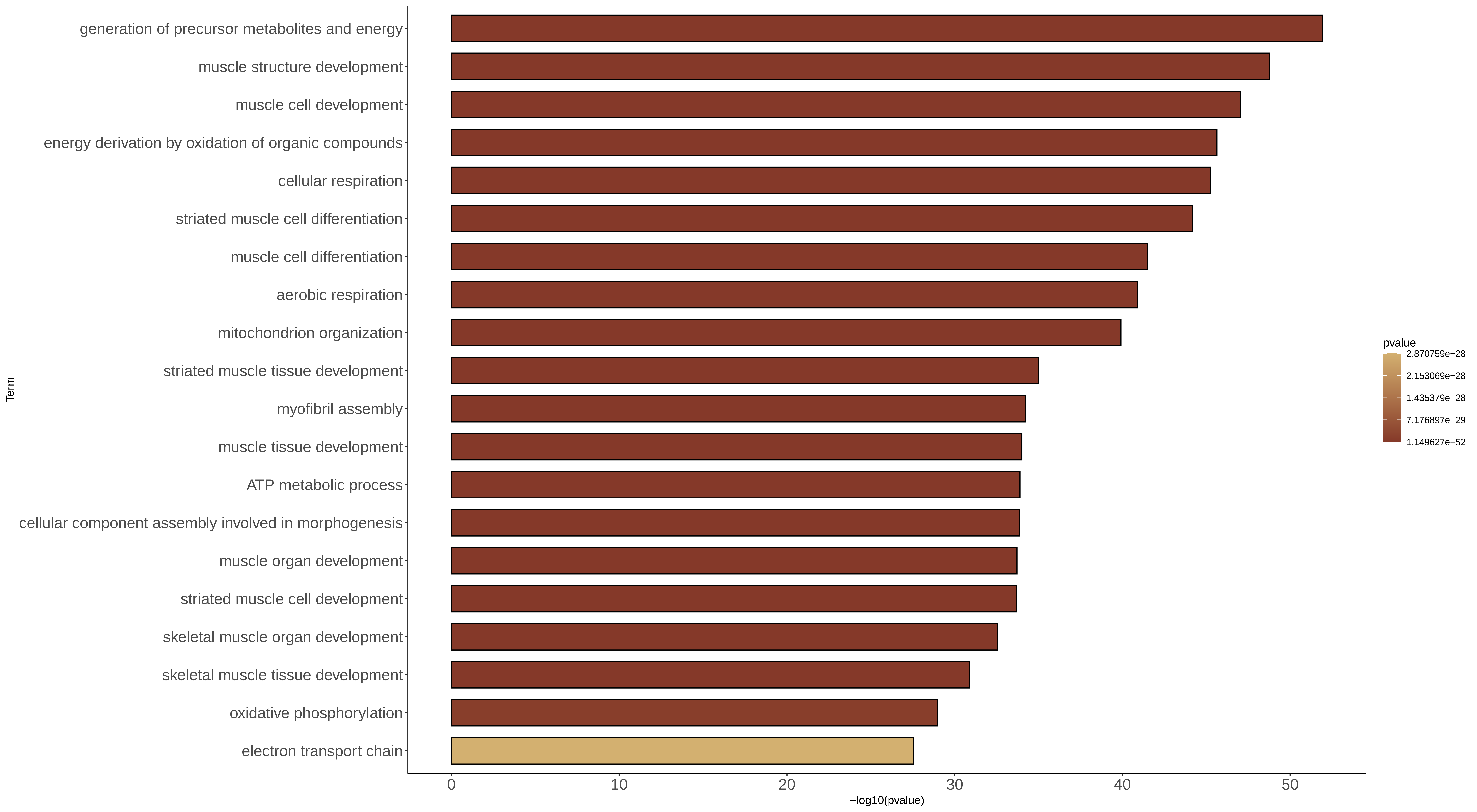
generation of precursor metabolites and energy
muscle structure development
muscle cell development
energy derivation by oxidation of organic compounds
cellular respiration
striated muscle cell differentiation
muscle cell differentiation
aerobic respiration
mitochondrion organization
striated muscle tissue development
myofibril assembly
muscle tissue development
ATP metabolic process
cellular component assembly involved in morphogenesis
muscle organ development
striated muscle cell development
skeletal muscle organ development
skeletal muscle tissue development
oxidative phosphorylation
electron transport chain
Term
0
10
20
30
40
50
−log10(pvalue)
pvalue
2.870759e−28
2.153069e−28
1.435379e−28
7.176897e−29
1.149627e−52
